# Top publications for advancing state and local health department antimicrobial stewardship programs

**DOI:** 10.1017/ash.2025.10172

**Published:** 2025-10-06

**Authors:** Lauren R. Biehle, Jamie Jacob, Tariq Mosleh, Tho Pham, Jenna Preusker, Galina Shteyman, Jessica Zering, Christopher D. Evans

**Affiliations:** 1 Colorado Department of Public Health and Environment, Denver, CO, USA; 2 Illinois Department of Public Health, Chicago, IL, USA; 3 Utah Department of Health and Human Services, Salt Lake City, UT, USA; 4 University of Arizona R. Ken Coit College of Pharmacy, Phoenix, Arizona, USA; 5 Nebraska Department of Health and Human Services, Lincoln, NE, USA; 6 Minnesota Department of Health, St. Paul, MN, USA; 7 Washington State Department of Health, Shoreline, WA, USA; 8 Tennessee Department of Health, Nashville, TN, USA

## Abstract

**Objective:**

Expanded public health (PH) antimicrobial stewardship (AS) funding to Healthcare-Associated Infections (HAI)/Antimicrobial Resistance (AR) programs led to hiring of pharmacists with AS expertise in health departments. A PH pharmacists’ community of practice (CoP) consisting of pharmacists who work for local, state, and federal PH agencies was initiated to share resources and activities from PH AS interventions and collaborations. The objective of this evaluation was to characterize publications identified by PH pharmacists’ CoP that helped advance AS implementation by PH agencies.

**Methods:**

In March 2024, a 34-item electronic questionnaire was developed and distributed to the PH pharmacists’ CoP requesting nominations of articles that highlight interventions and data sources essential to PH AS practice. Respondents were asked to submit 1–10 scientific articles published from 2014 to 2023 most relevant to their work in PH AS implementation and/or in achieving programmatic deliverables. Articles with ≥ 3 nominations were selected for review.

**Results:**

Of 39 PH pharmacists in the CoP, 24 (62%) responded with 106 article nominations. Respondents held state-level positions (83%), had <2 years of PH AS experience (54%), and were often from the southeastern United States (25%). Publications in acute care settings were most frequent (54, 51%), followed by outpatient (47, 44%), and long-term care (45, 42%). A total of eleven articles were selected for review.

**Conclusions:**

Scientific publications impactful to AS PH practice can serve as a valuable resource for AS programs and pharmacists in PH. Dissemination of PH AS activities can inform enhancement of AS programs in the PH sector.

## Introduction

Implementation of sustainable antimicrobial stewardship (AS) is essential to improve antibiotic use and combat resistance.^1^ The Centers for Disease Control and Prevention (CDC) recognized the importance of public health (PH) agencies in guiding AS and promoting appropriate antibiotic use by introducing the Core Elements of Antibiotic Stewardship for Health Departments in 2023 (CEs).^1^ Expanded PH AS funding to Healthcare-Associated Infections (HAI)/Antimicrobial Resistance (AR) programs led to the hiring of AS expertise in health departments. Pharmacists with experience implementing AS activities in healthcare settings lead or co-lead state and local health department (SLHD) AS programs. PH AS pharmacists are a novel specialty of pharmacists that can enhance population health by guiding and supporting stewardship activities and collaboratives with various partners, including integrated health systems, payers, professional organizations, academic partners, quality improvement organizations, and licensing and regulatory agencies (Table 1).

**Table 1.** Role of an antimicrobial stewardship pharmacist in public health

Partner(s)	Role of antimicrobial stewardship pharmacist in public health
**Clinician**	Provide individualized technical assistance in patient care settings (e.g., hospitals, dental clinics, outpatient clinics, dialysis centers, nursing homes) to improve AS implementation based on the Core Elements of Antibiotic Stewardship Analyze and disseminate state-level data to support clinicians with local benchmarking (e.g., National Healthcare Safety Network (NHSN) annual state survey data for acute and long-term care settings, prescriber-level feedback interventions) Assist in translation of NHSN Antimicrobial Use and Resistance (AUR) data into action at the local level Provide clinical decision-making support through the creation of guidance and education (e.g., guidance on the use of antibiotics or diagnostic testing, toolkits, educational webinars)
**Healthcare** **organization**	Promote best practices in AS through evaluation and guidance of initiatives described in the Core Elements of Antibiotic Stewardship Support One Health Collaborative strategies Create facility certificates (e.g., honor roll) to acknowledge success in implementing AS activities Host educational or training collaboratives to improve AS implementation and support survey readiness Participate in infection control assessment and response (ICARs) to assess practices and inform quality improvement Support acute care facilities with enrollment and submission of data to the NHSN’s AUR Module Provide funding opportunities to enhance the implementation of AS practices
**Regulatory or** **quality agency**	Serve as a subject matter expert for AS regulatory updates Collaborate with quality improvement networks/quality improvement organizations (QIN/QIO) to support AS efforts in under-resourced settings (e.g., long-term care, dialysis centers) Develop a strategic plan outlining short and long-term AS goals Explore collaboration with other health department activities to support AS (e.g., HAI surveillance, containment, etc.)
**General public**	Create and disseminate educational materials through social media and public-facing websites Perform educational outreach through community events

AS = antimicrobial stewardship, AUR = antimicrobial use and resistance, HAI = healthcare-associated infection, ICAR = infection control assessment and response, NHSN = National Healthcare Safety Network, QIN/QIO = quality improvement networks/quality improvement organizations.

In 2021, a network of PH pharmacists was created to share resources and foster collaboration. This PH pharmacists’ Community of Practice (CoP) consists of 39 pharmacists who work for local, state, and federal PH agencies. While literature exists to guide stewardship efforts in specific healthcare settings, there are no publications to support the unique role filled by the PH pharmacist. This evaluation aims to characterize high-impact publications identified by the PH pharmacists’ CoP that highlight valuable AS interventions in PH to educate clinicians, guide future initiatives, and encourage local discussion for further implementation.

## Methods

In March 2024, a 34-item electronic questionnaire (Supplemental Appendix I) was developed and distributed via Google Forms to the members of the PH pharmacists CoP (*n* = 39). The questionnaire requested nominations of scientific articles that highlight key interventions and data sources essential to PH AS practice. Respondents were asked to submit 1–10 articles most relevant to their work in PH AS implementation and/or in achieving programmatic deliverables. Eligible articles included clinical trials, meta-analyses, systematic reviews, and observational studies published between 2014 and 2023. Guidelines, abstracts, and guidance documents were excluded. The maximum age of publication was selected as 2014 as it correlated with the year that the CDC published the first Core Elements of Stewardship.^2^ Respondent demographics and nominated articles were characterized by overall count, geographical region, and healthcare setting. Articles with the highest number of nominations (≥ 3) were selected for review.

## Results

**Figure 1.**
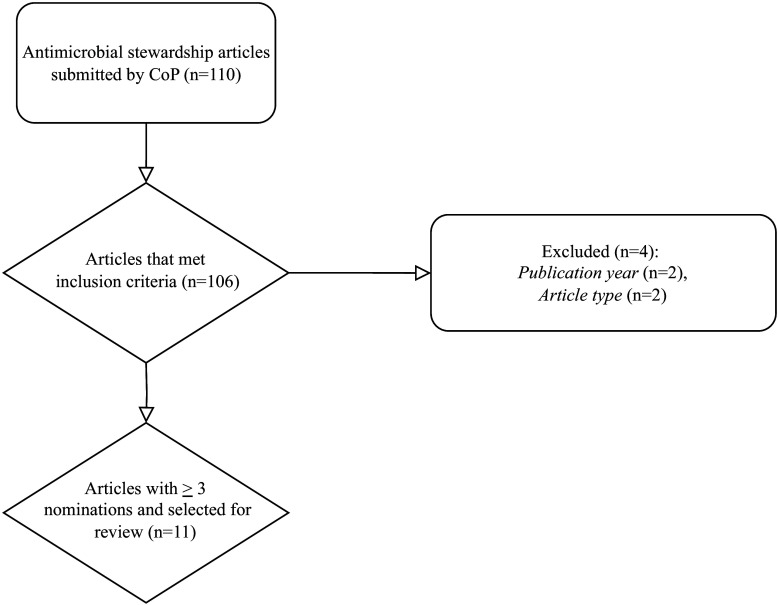
Flowchart of the article selection process.

Of 39 PH pharmacists serving in the CoP, 24 (62%) responded with a total of 110 article nominations and 106 nominations that met inclusion criteria (Figure 1). Most respondents held state-level positions (20, 83%) and had fewer than 2 years of PH AS experience (13, 54%). All geographical regions were represented, and the highest percentage of respondents were from the Southeastern (6, 25%) or Northeastern (4, 17%) regions of the United States (Table 2).

**Table 2.** Characteristics of survey respondents, 2024

Region of practice^*^	No, (%)
Central	3, (12.5)
Federal	2, (8.3)
Mid-Atlantic	1, (4.2)
Midwest	3, (12.5)
Mountain	3, (12.5)
Northeast	4, (16.7)
Southeast	6, (25)
West	2, (8.3)
**Respondent experience in public health practice**	
Less than 2 years	13, (54.2)
2 – 5 years	6, (25)
Greater than 5 years	5, (20.8)
**Antimicrobial stewardship is part of daily practice within a public health department/agency**	24, (100)
**Level of public health jurisdiction**	
Local (city, county)	2, (8)
State	20, (83)
Federal	2, (8)

* Region based on Antimicrobial Resistance Laboratory Networks.^3^

**Figure 2.**
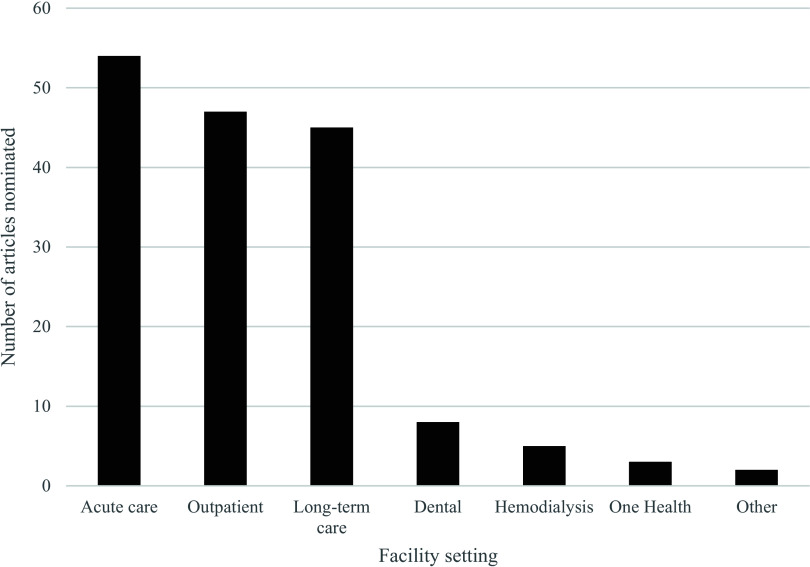
Number of articles nominated by facility setting.

The number of nominations per article ranged from one to eight. Due to an equal number of nominations for the tenth article, 11 articles were reported and summarized (Table 3). The highest number of articles was nominated by the Southeast (*n* = 29) and lowest by the Mid-Atlantic region (*n* = 4). Publications in acute care settings were most frequent (54, 51%), followed by outpatient (47, 44%), and long-term care (45, 42%) (Figure 2). Articles were categorized by respondents and may be applicable to multiple healthcare settings.

**Table 3.** Summary of most frequently nominated antimicrobial stewardship publications to advance public health

Study setting	Study citation	Study design	Results	N. of nominations (*n* = 24 respondents)
**Acute care**	Magill SS, et al Assessment of the appropriateness of antimicrobial use in US hospitals. *JAMA Netw Open.* 2021;4(3):e212007.	Cross-sectional prevalence survey	Study of random samples of 1566 patients at 192 hospitals Evaluated antimicrobial treatment for CAP and UTI; assessed use of fluoroquinolones and vancomycin Treatment unsupported for (876/1566) 56% of patients overall Treatment unsupported for 80% patients with CAP, 77% patients with UTI, 47% of fluoroquinolone use, 27% of vancomycin use Excessive duration (≥ 8 days) identified in 59% of patients with CAP Lack of documented signs and symptoms identified in 50% of patients with UTI	4
	O’Leary EN, et al An update from the NHSN on hospital antibiotic stewardship programs in the US, 2014–2021. *Open Forum Infectious Diseases.* 2024;11(2).	Cross-sectional analysis	Uptake of all 7 Core Elements increased from 2014 – 2021 (41% to 95%) Uptake of all 6 Priority Elements was 10% in 2021 AS listed in a contract or job description for 60% of program leaders Physician-pharmacist co-led programs increased from 23% to 64% Audit with feedback interventions reported in 76% of hospitals	3
	Winders HR, et al Show me the data: a statewide comparative report of NHSN Antimicrobial Use Option SAARs. *Antimicrob Steward Healthc Epidemiol*. 2022;2(1):e119.	Descriptive report	Description of South Carolina Comparative SAAR Analysis Reports from the NHSN AU option created from 2019 – 2021 Volume of facilities increased from average of 24 facilities/quarter in 2019 to 36 facilities/quarter in 2021	3
**Long-term care**	Kullar R, et al A roadmap to implementing antimicrobial stewardship principles in LTCFs: collaboration between an acute-care hospital and LTCFs. *Clin Infect Dis*. 2018;66(8):1304–1 312.	Guidance document	Key elements of ACH and LTCF partnerships include: Perform gap analysis Secure leadership support Develop program materials Establish systems for tracking outcomes and providing feedback Shared accountability	4
	Gouin KA, et al Implementation of core elements of antibiotic stewardship in nursing homes-National Healthcare Safety Network, 2016–2018. *Infect Control Hosp Epidemiol.* 2022;43(6):752–756.	Cross-sectional analysis	Uptake of all 7 Core Elements increased from 2016 – 2018 (43% – 71%) Increase in pharmacist involvement by 27% Facilities that reported minimum of 20 hours/week of infection prevention and control activities were 14% more likely to implement all 7 Core Elements	4
	Katz MJ, et al Implementation of an antibiotic stewardship program in LTCFs across the US. *JAMA Network Open.* 2022;5(2):e220181-e220181.	Before-and-after quality improvement initiative analysis	84% of enrolled LTCFs completed a 15 webinar series over 12 months Significant reduction in antibiotic starts by .41 per 1,000 resident-days was seen, with the greatest decrease in fluoroquinolone use Significant decrease in the number of urine cultures collected per 1,000 resident-days	4
**Outpatient**	Gouin KA, et al Identifying higher-volume antibiotic outpatient prescribers using publicly available Medicare Part D data - United States, 2019. *MMWR Morb Mortal Wkly Rep*. 2022;71(6):202–205.	Cross-sectional analysis	Median prescribing rate of the higher-volume prescribers was 60% higher than that of lower-volume prescribers Family practice and internal medicine most common specialties among the higher-volume prescribers Dentists had highest antibiotic prescribing rate of 1,271 prescriptions per 1,000 beneficiaries.	8
	Beshearse EM, et al Comparison of outpatient antibiotic prescriptions among older adults in IQVIA Xponent and publicly available Medicare Part D data, 2018. *Antimicrob Steward Healthc Epidemiol.* 2023;3(1):e32.	Cross-sectional analysis	Centers for Medicare and Medicaid Services (CMS) Part D Prescriber Public Use Files (PUFs) and IQVIA Xponent® have been used to track outpatient antibiotic use CMS Part D PUFs are available at no cost, while IQVIA Xponent® is associated with a fee Compared data suppression between datasets Distribution of antibiotic prescriptions across the datasets was comparable (< 2% variation) in region, antibiotic class, and prescriber specialty	4
	Kim CY, et al Characteristics of patients associated with any outpatient antibiotic prescribing among Medicare Part D enrollees, 2007–2018. *Antimicrob Steward Healthc Epidemiol*. 2023;3(1):e113.	Cross-sectional analysis	Linked National Health Interview Survey data linked with Medicare Part D antibiotic prescription claims Patient demographic, health-related, geographic, and socioeconomic characteristics were associated with variations in receipt of antibiotic prescriptions Part D beneficiaries with any antibiotic prescription claims were overall more likely to live in urban areas, to be female, to be non-Hispanic White, to have self-reported excellent or very good/good health status, and to have ≤ 3 chronic conditions	4
	Kim C, et al Health equity and antibiotic prescribing in the US: a systematic scoping review. *Open Forum Infect Dis*. 2023;10(9):ofad440.	Scoping review	Review of articles from 2000 – 2022 to characterize inequities in antibiotic prescribing and use 61 studies included, with 90% from the outpatient setting Antibiotic differences in prescribing found by patient’s race, ethnicity, sex, age, socioeconomic factors, geography, clinician age and specialty, and healthcare setting	4
	Stenehjem E, et al Implementation of an Antibiotic Stewardship Initiative in a Large Urgent Care Network. *JAMA Netw Open*. 2023;6(5):e2313011.	Before-and-after quality improvement initiative analysis	38 urgent care and 1 telemedicine clinic participated in an initiative reducing antibiotic prescribing for respiratory conditions AS interventions included education for clinicians and patients, electronic health record tools, clinician benchmarking dashboard, and media Antibiotic prescribing was reduced by 15% for respiratory conditions (48% to 33%) The antibiotic prescribing rate remained lower than baseline during the sustainability period	4

ACH = acute-care hospital, AS = antimicrobial stewardship, AU = antimicrobial use, CAP = community-acquired pneumonia, CMS = Centers for Medicare and Medicaid Services, LTCF = long-term care facility, NHSN = National Healthcare Safety Network, PUF = public use file, SAAR = Standardized Antimicrobial Administration Ratio, US = United States of America, UTI = urinary tract infection.

The most frequently nominated articles by respondents (Table 3) were categorized into the settings of acute care (*n* = 3), long-term care (*n* = 3), and outpatient (*n* = 5). The article with the most nominations (eight) is a publication identifying high-volume antibiotic outpatient prescribers for a state-led audit and feedback intervention. Articles are summarized and presented below by facility type or care setting. An expanded list of nominated articles can be found in Supplemental table 1.

### Acute care

In 2014, the CDC released the Core Elements (CE) of Hospital Antibiotic Stewardship Programs (ASP) to outline best practices for optimizing antibiotic use in the inpatient care setting.^2^ The articles nominated for the acute care setting describe common themes of inpatient antibiotic use and implementation of the core elements.

In the 2023 article by O’Leary et al, the authors describe the uptake of the CE by hospitals throughout the U.S. from 2014 o 2021 based on responses to the National Healthcare Safety Network (NHSN) Annual Hospital Survey.^4^ Over 4,000 hospitals completed the survey each year and uptake of all CE increased from 41% to 95% between 2014 and 2021. The most widely implemented intervention was a policy/procedure for optimizing the treatment of sepsis (81%) and the least common was the review of outpatient parenteral antibiotic therapy (22%).^4^ In 2022, CDC released the Priorities for Hospital CE Implementation to highlight a subset of approaches that are highly effective.^5^

In addition to the NHSN Annual Hospital Survey, the NHSN Antimicrobial Use and Resistance Module provides data for tracking and reporting in the acute care setting. The Standardized Antimicrobial Administration Ratio (SAAR) is a risk-adjusted summary measure that compares observed to predicted antimicrobial days for groups of antimicrobials. In the article by Winders et al., the authors describe quarterly Comparative SAAR Analysis Reports created by the Antimicrobial Stewardship Collaborative of South Carolina (SC) and the SC Department of Health and Environmental Control.^6^ These reports provide SAAR histograms and site-specific feedback to participating facilities in their state. Facilities reported generally positive feedback regarding these reports and inclusion of the reports into their ASP.

Prior assessments of antibiotic appropriateness in the acute setting have reported approximately 30% suboptimal or unnecessary antibiotic prescribing.^7–9^ The 2015 cross-sectional prevalence survey conducted by Magill et al.^7^ expanded on previous hospital surveys executed by Emerging Infections Program staff to examine the quality of antimicrobial prescribing in a random sample of inpatients receiving treatment for community-acquired pneumonia (CAP) and urinary tract infection (UTI). They also assessed the prescribing of two select antimicrobials: fluoroquinolones and intravenous vancomycin.^7^ The results demonstrated a substantial percentage of treatment in all four categories was unsupported. The most common reasons for unsupported use included long duration (103/174 [59%] in CAP), treatment deviation from guidelines (68/174 [39%] in CAP), lack of documented signs or symptoms of infection (174/347 [50%] in UTI), and lack of microbiologic evidence of infection (95/347 [27%] in UTI).

These acute care articles highlight the advancements and areas of opportunity for hospitals implementing the core elements and optimizing antibiotic use. Pharmacists supporting stewardship and PH can utilize the information from these studies to direct hospital stewardship programs to engage in the establishment of initiatives that are effective and target areas of unsupported antibiotic use. Providing SAAR benchmarking by region and/or bed size is a unique role that can be fulfilled by SLHD stewardship programs.

### Long-term care

The Core Elements of Antibiotic Stewardship for Nursing Homes were released in 2015.^10^ In 2016, the Centers for Medicare and Medicaid Services (CMS) finalized a rule requiring nursing homes to implement an ASP, including antibiotic use protocols and systems for monitoring antibiotic use.^11^ Although the framework for long-term care facility (LTCF) ASPs aligns with those developed for hospitals, LTCFs face distinctive challenges in implementation, presenting an excellent opportunity for collaboration with PH AS pharmacists to ensure robust ASPs in this setting.

Gouin et al conducted a retrospective, repeated cross-sectional analysis of 7,506 annual surveys from the NHSN LTCF Component to assess the implementation of the CDC Core Elements (CE) of Antibiotic Stewardship in Nursing Homes from 2016 to 2018.^12^ In 2018, 71% of nursing homes had implemented all seven CE, a 28% increase from 2016, with significant improvements in education (19% increase from 2016 to 2018), reporting (18%), and drug expertise (15%). Notably, there was a 27% increase in pharmacist involvement, with 71% of nursing homes reporting pharmacist participation in 2018. Facilities reporting at least 20 hours per week of infection prevention and control activities were 14% more likely to implement all seven CE when controlling for facility ownership and affiliation. Overall, nursing homes reported substantial progress in AS implementation from 2016 to 2018, driven in part by increased regulatory requirements and enhanced access to expertise and resources.

Katz MJ et al evaluated the Agency for Healthcare Research and Quality (AHRQ) Safety Program for Improving Antibiotic Use in LTCFs across the U.S. from 2018 to 2019.^13^ This quality improvement initiative aimed to establish ASPs in LTCFs by addressing both facility culture and technical knowledge related to antibiotic prescribing. Fifteen webinars occurred over 12 months, accompanied by tools, activities, posters, and pocket cards to support education and implementation. Of 523 LTCFs recruited, 439 (84%) completed the program. The results demonstrated a significant reduction in antibiotic starts by 0.41 per 1,000 resident-days, with the greatest decrease in fluoroquinolone use. While the overall reduction in days of antibiotic therapy (DOT) was not significant, facilities with greater program engagement saw notable decreases in both antibiotic starts and DOT. In addition, there was a significant decrease in the number of urine cultures per 1 000 resident-days. Participation in the program was associated with the development of ASPs that actively engaged clinical staff in the decision-making processes around antibiotic prescriptions in participating LTCFs. The reduction in antibiotic starts and DOT in engaged facilities underscores the potential of a multifaceted program, including patient-safety principles, multidisciplinary education, and interactive tools, to support successful ASPs in LTCFs.

Kullar et al provides a practical roadmap for implementing comprehensive ASPs in LTCFs by leveraging the established expertise and infrastructure of acute care hospitals (ACHs) to address common challenges faced by LTCFs.^14^ This partnership has proven beneficial in reducing rates of multidrug-resistant organisms and *Clostridioides difficile* infections and ensuring compliance with regulatory requirements.^15,16^ The roadmap outlines several key elements: performing a gap analysis of LTCF AS practices, securing LTCF and ACH leadership support, developing and implementing program materials, and establishing systems for measurement and feedback of antibiotic use and outcomes. Furthermore, the article highlights the importance of overcoming barriers, such as limited resources and expertise, educational gaps, public and patient expectations, and distinguishing between colonization and infection. Shared accountability fosters stability and continuity in the implementation and maintenance of ASPs, highlighting the importance of collaboration in establishing effective and sustainable ASPs in LTCFs.

The reviewed publications collectively indicate that significant progress has been made in LTCF ASPs, with notable improvements in AS practices and outcomes. However, the studies also reveal persistent gaps and disparities in implementation, particularly concerning resource availability and staff engagement. Future efforts should prioritize sustaining and expanding these gains by ensuring adequate resources, fostering strong inter-organizational collaborations, and continuing to engage and educate LTCF staff. Further research is needed to explore innovative strategies for overcoming the unique challenges faced by LTCFs, such as high staff turnover and the complex healthcare needs of residents. This includes exploring new models of care, improving data collection and analysis, and expanding communication and collaboration between LTCFs and other healthcare sectors. PH AS pharmacists play a pivotal role in addressing these gaps by providing expertise, support, and a framework for continuous improvement.

### Outpatient

Many AS efforts have been directed toward acute care; yet, most of antibiotics are prescribed in the outpatient setting.^17^ Optimizing antibiotic prescribing in the outpatient setting remains challenging due to limited resources, lack of continuum of care, and the differences of healthcare professionals and patient populations.^18,19^

Tracking antibiotic use data can identify prescribing patterns and key drivers of antibiotic use.^20^ Gouin et al.^21^ compared antibiotic prescribing rates between lower- and higher-volume prescribers (the highest 10^th^ percentile of prescriber-level antibiotic volume) by specialty and geography using the 2019 CMS Part D Prescriber Public Use Files^22^ (PUFs). Of 59 million antibiotic prescriptions and 697,065 prescribers, 24 million (41%) were prescribed by the higher-volume prescribers (*n* = 69,835). The median prescribing rate of the higher-volume prescribers was 60% higher than that of lower-volume prescribers (*P* < .001). Predominantly practicing in the South, the higher-volume prescribers wrote 12.3 million (49%) antibiotic prescriptions in the region and had the highest antibiotic prescribing rate (696 prescriptions per 1,000 beneficiaries). Family practice and internal medicine were the most common specialties among the higher-volume prescribers, accounting for more than 20% of total antibiotic volume. Dentists accounted for only 3% of higher-volume prescribers but had the highest antibiotic prescribing rate of 1,271 prescriptions per 1,000 beneficiaries. The publicly available CMS Part D PUFs are valuable resources for healthcare organizations to identify opportunities for antibiotic stewardship in the outpatient setting.

Other proprietary data sources such as IQVIA Xponent^®23^ have also been used to track outpatient antibiotic use. CMS Part D PUFs capture approximately 70% of Medicare beneficiaries. IQVIA Xponent® is projected to capture 100% of antibiotic prescriptions filled in U.S. retail pharmacies.^24^ While CMS Part D PUFs are available at no cost to the public, IQVIA Xponent® is associated with a fee. Understanding the limited resources and funding that PH organizations are facing, Beshearse et al evaluated and compared prescription data between CMS Part D PUFs and IQVIA Xponent® among adults ≥ 65 years. ^25^ The distribution of antibiotic prescriptions across the datasets were comparable (<2% variation) in terms of region, antibiotic class, and prescriber specialty. Neither data set offers detailed clinical diagnoses nor indications; thus, the appropriateness of antibiotic use cannot be assessed. CMS Part D PUFs does have a 2-year data lag and suppression of prescription claim counts <11 for confidentiality, which may impact prescribing patterns.

Due to the differences of patient populations in the outpatient setting, a deeper understanding of patient sociodemographic and baseline health characteristics can improve antibiotic prescribing among vulnerable populations and reduce health disparities.^26^ Kim et al assessed the associations of patient demographic, health-related, geographic, and socioeconomic characteristics with the receipt of antibiotics using a logistic regression model.^27^ Patients were more likely to receive antibiotics if they were female (1.31; 95% CI 1.21–1.48), residing in the South (1.18; 95% CI 1.03–1.36), and reporting inability to afford prescription drugs (1.41; 95% CI, 1.11–1.79). Having one or more chronic comorbidities and being non-Hispanic White were also associated with antibiotic receipt. The study highlights the variations in receipt of antibiotic prescriptions and warrants further investigation of antibiotic prescribing disparities.

As the healthcare landscape is constantly evolving, patients are leaning toward non-traditional outpatient settings such as urgent care (UC) clinics due to lower costs and convenience.^18,28^ However, compared to other outpatient settings, UCs have higher antibiotic use underscoring the importance of implementing effective antibiotic stewardship initiatives.^29^ Stenehjem et al conducted a before-and-after quality improvement program at Intermountain Health UC network consisting of 38 UC clinics and one telemedicine clinic to reduce antibiotic prescribing for respiratory conditions (otitis media, sinusitis, and pharyngitis).^30^ The interventions included UC clinician and patient education, electronic health record tools for clinician ordering and documentation, a clinician benchmarking dashboard, and media targeting patients and clinicians. Each site had a 12-month baseline period, a 12-month intervention period, and a 12-month sustainability period. There were 207,047 and 183,893 UC encounters in the baseline and intervention periods, respectively. Overall, antibiotic prescribing was reduced by 15% for respiratory conditions. Despite a 22% reduction in the initial month, antibiotic prescribing continued to reduce by 5% each month during the intervention period across clinic types and clinicians (*P* < .001). The antibiotic prescribing rate remained lower than baseline during the sustainability period. In addition to the decrease in prescribing, the initiative also improved antibiotic selection for the respiratory conditions.

If identifying PH data sources of antibiotic use is challenging, identifying sources that contain demographics suitable for health equity analyses is even harder. In 2023, Kim et al performed a scoping review of literature describing inequities in antibiotic prescribing from 2000 to 2022.^31^ They included 61 articles in their review; this article did review other settings, but we include it in this setting because 55 of the 61 were from the outpatient setting. Other settings included: three from dentistry, two from long-term care, and a single article from acute care. ^31^ Most of the studies did not have health equity as a primary objective, but rather assessed overall antibiotic prescribing and factors associated with receiving antibiotics through multivariate modeling. ^31^ Characteristics that were associated with receiving more antibiotics included: age (age < 5 yr and older adults), female gender, white race, non-Hispanic ethnicity, having private insurance, high socioeconomic status, being seen by an advanced practice provider, or living in the South census region or a rural setting. ^31^ Characteristics associated with receiving fewer antibiotics included Black race or Hispanic ethnicity, being seen in the emergency department, or being seen by a pediatrician. ^31^ While most PH messaging around antibiotic prescribing aims to decrease overall numbers of prescriptions, it should not be inferred that Black or Hispanic persons receive higher quality care. In fact, while Black children are 25% less likely to receive an antibiotic from the same clinician and 12% less likely to receive a broad-spectrum antibiotic than non-Black children, they are also 28% less likely to receive antibiotics for a respiratory infection when they are clinically indicated.^32,33^ No articles in this review assessed gender identity, sexual orientation, immigrant or refugee status, disability, or homelessness.^31^ These characteristics are markers of antibiotic prescribing disparities, but it is the drivers behind them which may truly influence prescribing patterns.^31^ For example, while age and race are markers of antibiotic prescribing disparities, they are driven by implicit bias and structural racism within the healthcare community and/or its prescribers.^31^ Recognizing these underlying drivers is essential for developing stewardship interventions that promote equity in antibiotic prescribing.

In conclusion, the highlighted studies underscored the importance of tracking antibiotic use, analyzing prescribing patterns, identifying opportunities for antibiotic stewardship implementation, and the impact on prescribing rate and optimizing antibiotic selection in the outpatient setting.

## Discussion

This review identified practical and impactful AS PH articles that can help PH AS pharmacists to advance AS initiatives in different health care settings and ultimately improve patient outcomes. It builds upon previous work authored by the Baker’s Dozen^34^ and Houston groups,^35^ which aimed to provide clinicians with a summary of the most impactful AS-focused research. While these focused on specific settings or disease states, this review covers a broader range of AS in different healthcare settings. It emphasizes population health rather than individual health with the most impactful articles selected being those that summarized antibiotic prescribing and AS implementation in large databases or samples. This review offers a comprehensive resource for PH AS programs seeking to partner with clinicians and meet the CEs for HDs.

While the selected articles discussed AS progress, they also underscored the need for PH pharmacists to provide expertise and support the sustainability of AS initiatives. Healthcare facilities with dedicated AS resources were also more likely to successfully implement the CEs and improve patient care. Hence, AS PH pharmacists are in a unique position to address these challenges. Clinicians in any healthcare setting can benefit from this free and accessible PH expertise, particularly organizations with limited resources (eg, critical access hospitals and nursing homes) (Table 1). Direct engagement with PH AS expertise can be initiated through electronic means, local health officers, state-level meetings, or AS-run collaboratives.

This is the first review article to exclusively focus on articles in PH AS. These publications capture the timeline and external validity of CE implementation and are inclusive of the COVID-19 pre- and postpandemic periods. They represent different healthcare settings and novel perspectives from a unique AS role. However, some limitations exist. The surveyed group was small at 39 AS PH pharmacists. Nonetheless, the response rate was 62% (24/39) and respondents were geographically diverse. The date range of the nominated articles was broad due to a low number of recent publications. Despite this, over 100 articles in total were nominated (Supplemental Table 1). Future work could utilize modified Delphi methodology to further refine article selection. Few nominated articles focused directly on implementation of the CEs for SLHDs; however, these were released in 2023. More research and data informing the application of these are needed and will inform future iterations of this review.

## Supporting information

10.1017/ash.2025.10172.sm001 Biehle et al. supplementary material Biehle et al. supplementary material

## Supplementary material

The supplementary material for this article can be found at https://doi.org/10.1017/ash.2025.10172.
