# Supplementary material for: Top publications for advancing state and local health department antimicrobial stewardship programs
Source: Antimicrob Steward Healthc Epidemiol. 2025 Oct 6;5(1):e249. doi: 10.1017/ash.2025.10172 (PMC12509152; doi:10.1017/ash.2025.10172)
Supplement: Biehle et al. supplementary material [file S2732494X25101721sup001.docx]

Appendix I (Supplemental). Survey instrument.

Survey title: Top Publications for Advancing Public Health Antimicrobial Stewardship Practices

1. Is antimicrobial stewardship part of your daily practice within a public health department/agency?
   1. Yes
   2. No
2. In what level of public health jurisdiction do you currently practice?
   1. Local (city/county)
   2. State
   3. Federal
3. Which region of the United States do you practice in? Regions listed correspond to the Antimicrobial Resistance Laboratory Network.
   1. Northeast
   2. Mid-Atlantic
   3. Southeast
   4. Midwest
   5. Central
   6. Mountain
   7. West
   8. U.S. Territories
   9. Federal
   10. Prefer not to answer
4. Please indicate your time of work experience in public health practice.
   1. Less than 2 years
   2. 2-5 years
   3. Greater than 5 years
5. Publications 1-10: Please list up to 10 top publications essential to your public health antimicrobial stewardship practice. Please include 1 publication per line. You do not need to rank the publications in any particular order.
6. Publications 1-10: Please select an option for the application of the publication(s). Select all that apply.
   1. Article for functioning as a public health stewardship pharmacist
   2. Influential publication to achieve public health stewardship deliverables
7. Publications 1-10: Please select the healthcare setting that would benefit most from the publication(s).
   1. Acute care
   2. Long-term care
   3. Outpatient (including primary care, urgent care, emergency department, etc)
   4. Dental
   5. Hemodialysis
   6. One Health (veterinary, agriculture, etc)
   7. Not applicable

Table 1 (Supplemental). Articles with two or more nominations from the Community of Practice, listed by setting and number of nominations.

| **Study Setting** | **Study Citation** | **N. of nominations (n=24 respondents)** |
| --- | --- | --- |
| **Acute Care** | | |
|  | O'Leary EN, Neuhauser MM, McLees A, Paek M, Tappe J, Srinivasan A. An update from the National Healthcare Safety Network on hospital antibiotic stewardship programs in the United States, 2014-2021. *Open Forum Infect Dis* 2024;11(2):ofad684. doi: 10.1093/ofid/ofad684 | 3 |
|  | Winders HR, Antosz K, Al-Hasan M, et al. Show me the data: a statewide comparative report of National Healthcare Safety Network (NHSN) Antimicrobial Use Option standardized antimicrobial administration ratios (SAARs). *Antimicrob Steward Healthc Epidemiol* 2022;2(1):e119. doi: 10.1017/ash.2022.266 | 3 |
|  | Curran J, Lo J, Leung V, et al. Estimating daily antibiotic harms: an umbrella review with individual study meta-analysis. *Clin Microbiol Infect* 2022;28(4):479-490. doi: 10.1016/j.cmi.2021.10.022 | 2 |
|  | Kim C, Kabbani S, Dube WC, et al. Health equity and antibiotic prescribing in the United States: a systematic scoping review. *Open Forum Infect Dis* 2023;10(9):ofad440. doi:10.1093/ofid/ofad440 | 4 |
|  | Kullar R, Yang H, Grein J, Murthy R. A roadmap to implementing antimicrobial stewardship principles in long-term care facilities (LTCFs): collaboration between an acute-care hospital and LTCFs. *Clin Infect Dis* 2018;66(8):1304-1312. doi:10.1093/cid/cix1041 | 2 |
|  | Magill SS, O'Leary E, Ray SM, et al. Assessment of the appropriateness of antimicrobial use in US Hospitals. *JAMA Netw Open* 2021;4(3):e212007. doi:10.1001/jamanetworkopen.2021.2007 | 2 |
|  | Mendelson M, Morris AM, Thursky K, Pulcini C. How to start an antimicrobial stewardship programme in a hospital. *Clin Microbiol Infect* 2020 ;26(4):447-453. doi: 10.1016/j.cmi.2019.08.007 | 2 |
|  | O'Leary EN, Edwards JR, Srinivasan A, et al. National Healthcare Safety Network Standardized Antimicrobial Administration Ratios (SAARs): a progress report and risk modeling update using 2017 data. *Clin Infect Dis* 2020 ;71(10):e702-e709. | 2 |
|  | van Santen KL, Edwards JR, Webb AK, et al. The Standardized Antimicrobial Administration Ratio: a new metric for measuring and comparing antibiotic use. *Clin Infect Dis* 2018;67(2):179-185. doi: 10.1093/cid/ciy075 | 2 |
| **Outpatient** | | |
|  | Gouin KA, Fleming-Dutra KE, Tsay S, Bizune D, Hicks LA, Kabbani S. Identifying higher-volume antibiotic outpatient prescribers using publicly available Medicare Part D data - United States, 2019. *MMWR Morb Mortal Wkly Rep* 2022;71(6):202-205. doi:10.15585/mmwr.mm7106a3 | 8 |
|  | Stenehjem E, Wallin A, Willis P, et al. Implementation of an antibiotic stewardship initiative in a large urgent care network. *JAMA Netw Open* 2023;6(5):e2313011. doi:10.1001/jamanetworkopen.2023.13011 | 4 |
|  | Kim C, Kabbani S, Dube WC, et al. Health equity and antibiotic prescribing in the United States: a systematic scoping review. *Open Forum Infect Dis* 2023;10(9):ofad440. doi:10.1093/ofid/ofad440 | 4 |
|  | Beshearse EM, Gouin KA, Fleming-Dutra KE, Tsay S, Hicks LA, Kabbani S. Comparison of outpatient antibiotic prescriptions among older adults in IQVIA Xponent and publicly available Medicare Part D data, 2018. *Antimicrob Steward Healthc Epidemiol* 2023;3(1):e32. doi:10.1017/ash.2022.332 | 2 |
|  | Curran J, Lo J, Leung V, et al. Estimating daily antibiotic harms: an umbrella review with individual study meta-analysis. *Clin Microbiol Infect* 2022;28(4):479-490. doi: 10.1016/j.cmi.2021.10.022 | 2 |
|  | Kim CY, Gouin KA, Hicks LA, Kabbani S. Characteristics of patients associated with any outpatient antibiotic prescribing among Medicare Part D enrollees, 2007-2018. *Antimicrob Steward Healthc Epidemiol* 2023;3(1):e113. doi:10.1017/ash.2023.180 | 2 |
| **Long-term care** | | |
|  | Adre C, Jump RLP, Spires SS. Recommendations for improving antimicrobial stewardship in long-term care settings through collaboration. *Infect Dis Clin North Am* 2020;34(1):129-143. doi: 10.1016/j.idc.2019.10.007 | 2 |
|  | Curran J, Lo J, Leung V, et al. Estimating daily antibiotic harms: an umbrella review with individual study meta-analysis. *Clin Microbiol Infect* 2022;28(4):479-490. doi: 10.1016/j.cmi.2021.10.022 | 2 |
|  | Katz MJ, Tamma PD, Cosgrove SE, et al. Implementation of an antibiotic stewardship program in long-term care facilities across the US. *JAMA Netw Open* 2022;5(2):e220181. doi:10.1001/jamanetworkopen.2022.0181 | 2 |
|  | Kullar R, Yang H, Grein J, Murthy R. A roadmap to implementing antimicrobial stewardship principles in long-term care facilities (LTCFs): collaboration between an acute-care hospital and LTCFs. *Clin Infect Dis* 2018;66(8):1304-1312. doi:10.1093/cid/cix1041 | 2 |
|  | Morrill HJ, Caffrey AR, Jump RL, Dosa D, LaPlante KL. Antimicrobial stewardship in long-term care facilities: a call to action. *J Am Med Dir Assoc* 2016;17(2):183.e1-16. doi: 10.1016/j.jamda.2015.11.013 | 2 |
| **Dental** | | |
|  | Suda KJ, Calip GS, Zhou J, et al. Assessment of the appropriateness of antibiotic prescriptions for infection prophylaxis before dental procedures, 2011 to 2015. *JAMA Netw Open* 2019;2(5):e193909. doi: 10.1001/jamanetworkopen.2019.3909 | 2 |
| **Hemodialysis** | | |
|  | Apata IW, Kabbani S, Neu AM, et al. Opportunities to improve antibiotic prescribing in outpatient hemodialysis facilities: a report from the American Society of Nephrology and Centers for Disease Control and Prevention Antibiotic Stewardship White Paper Writing Group. *Am J Kidney Dis* 2021 ;77(5):757-768. doi: 10.1053/j.ajkd.2020.08.011 | 2 |
|  | Hahn PD, Figgatt M, Peritz T, Coffin SE. Inappropriate intravenous antimicrobial starts: An antimicrobial stewardship metric for hemodialysis facilities. *Infect Control Hosp Epidemiol* 2019;40(10):1178-1180. doi: 10.1017/ice.2019.219. | 2 |
